# The Role of PRLR Gene Polymorphisms in Milk Production in European Wild Rabbit (*Oryctolagus cuniculus*)

**DOI:** 10.3390/ani13040671

**Published:** 2023-02-15

**Authors:** Ildikó Benedek, Vilmos Altbäcker, Attila Zsolnai, István Nagy, Dávid Mezőszentgyörgyi, Tamás Molnár

**Affiliations:** 1 Department of Animal Breeding, Institute of Animal Breeding Sciences, Hungarian University of Agriculture and Life Sciences, 7400 Kaposvar, Hungary; 2 Department of Nature Conservation, Institute for Wildlife Management and Nature, Hungarian University of Agriculture and Life Sciences, 7400 Kaposvar, Hungary; 3 Department of Molecular Ecology, Institute of Aquaculture and Environmental Safety, Hungarian University of Agriculture and Life Sciences, 7400 Kaposvar, Hungary

**Keywords:** prolactin receptor gene, milk production, SNP, microsatellite, *Oryctolagus cuniculus*, wild rabbit

## Abstract

**Simple Summary:**

In rabbits, milk is the primary source of nutrition from early growth to weaning. The ability of the mother rabbit to produce milk, which is also influenced by the maternal genotype, is particularly important in the case of the larger litters. The hormone prolactin is responsible for the initiation and maintenance of lactation and for the synthesis of the major components of milk. Prolactin acts through membrane receptors in target tissues. Point mutations and microsatellites in receptor genes can affect production characteristics. Our aim was to examine the prolactin receptor gene in a wild rabbit (*Oryctolagus cuniculus*) population with a diverse genetic background. Our hypothesis was that the detected polymorphisms could be associated with milk production. By sequencing the promoter region of the PRLR gene, we detected four point mutations and one microsatellite. Among the genotypes of point mutations in the regulatory region of the PRLR gene, the homozygous genotype and the short repeat of the microsatellite resulted in higher milk production. These could be potential marker candidates for the development of marker-assisted selection.

**Abstract:**

One of the problematic points of rabbit breeding is that the nutritional requirements of the kits are not fully satisfied by the does’ milk production from the third week of lactation onwards. The prolactin receptor gene has a significant effect on reproductive processes, and its polymorphisms have been associated with milk production in several species (cattle, goats, sheep, and buffalo). The European wild rabbit (*Oryctolagus cuniculus*), has a more diverse genetic background compared to domesticated lines. In the course of our study, sequencing of the 1210 bp long segment of the PRLR gene promoter region was accomplished. We detected four point mutations (SNP1-407G > A, SNP2-496G > C, SNP3-926T> and SNP4-973A > C) and one microsatellite at position 574. In our population, the four SNPs were segregated into four genotypes: AACCCCCC, GGGGTTAA, AAGGTTAC, and GGGGTCAC. Our results show that the genotype in the homozygous form is associated with higher milk production (1564.7 ± 444.7 g) compared to the other three genotypes (AACCCCCC 1399.1 ± 326.8 g; GTGACCTT 1403.8 ± 517.1 g; GGGGTCAC 1220.0 ± 666.2 g), and the short microsatellite repeat (167 bp) also coincides with significantly higher milk production (1623.8 ± 525.1 g). These results make the marker-assisted selection possible also for domesticated lines.

## 1. Introduction

From the early growth stage until the time of weaning, milk is the only nutrient source available to the kits and they are dependent on the doe’s milk production [1]. The milk production potential of the rabbit doe is well characterized by the survival, growth rate and weight of the litter at weaning [2]. However, from the third week of lactation, the doe is not able to produce sufficient milk to satisfy the nutrient requirements of the kits [3]. Among other factors (e.g., nutrition [4,5], parity, stage of lactation [6], litter size [7], physiological status [8], the seasonal effect [9]), maternal milk production is predominantly influenced by maternal genotype [10,11,12].

In domestic animals, the identification of genes associated with different complex traits began in the 1990s, identifying candidate genes and molecular variants associated with different phenotypic traits (genome-wide association studies (GWAS)) [13]. Studies on the variability of the rabbit genome [14,15,16] showed that the European wild rabbit is one of the most polymorphic mammals (nucleotide diversity ranges from 0.6 to 0.9), and therefore, provides an excellent model to explore the genetic background of production traits.

At the end of pregnancy, increasing oestradiol and decreasing progesterone levels coincide with increasing prolactin (PRL) and oxytocin hormone levels, while lactation is characterized by lower oestradiol, absent progesterone and high oxytocin and PRL concentrations [17]. PRL, through its effects in the central nervous system and peripheral tissues, affects a number of physiological functions, through its effects in the central nervous system and peripheral tissues, PRL affects a wide range of physiological functions, which can be grouped into several categories: (1) water and electrolyte balance, (2) growth and development, (3) endocrinology and metabolism, (4) brain and behavior, (5) reproduction, and (6) immune regulation and defense [18]. It is generally accepted that its role is essential for the initiation and maintenance of lactation and that it is primarily responsible for the synthesis of the major components of milk, milk proteins, lactose and fats [19]. The prolactin receptor gene (PRLR) plays an important role in the PRL signaling cascade [20].

PRL exerts its function in target tissues through its membrane receptors [21]. PRL hormone levels increase at the end of gestation (stimulated by a decrease in estrogen), and the RNA expression of PRL receptors increases 4-10-fold by the day of parturition [22]. PRLR seems to be a really promising one because it affects not only reproductive and growth traits but also milk production characteristics [23]. In different cattle breeds (*Bos taurus*), several point mutations in the PRLR gene have been found to be linked to differences in milk production [20,24,25], affecting milk yield and milk fat content. Similar results were reported in buffaloes (*Bubalus bubalis*) [26,27] and in several goat breeds (*Capra aegagrus hircus*) [28,29]. However, not only point mutations can affect production traits, but also microsatellite length polymorphisms too, as the number of repeats can be significant for gene expression and expression level [30]. Microsatellites may participate in the regulation of transcription when they are located in intragenic regions (promoters, 5′- and 3′-untranslated regions, and introns), and therefore, represent an important source of variation in quantitative traits (such as milk production) [31,32,33].

Although different factors influence milk production in rabbits, milk production curves differ among breeds [8,34,35], which shows the importance of genetic background. Milk yield and milk composition of crossbred lines can be related to the proportion of maternal genotype [35]. As polymorphisms in the PRLR gene have been associated with milk production in other species, we aimed to investigate the PRLR gene in a European Wild rabbit population (*Oryctolagus cuniculus*) where the genetic background was more variable compared to domesticated lines. Our hypothesis was that the PRLR gene of the European wild rabbit may contain several variants, among which polymorphisms could be found associated with milk production capacity.

## 2. Materials and Methods

### 2.1. Ethical Approval

The research was approved by the Committee on the Ethics of Animal Experiments of the Kaposvár Campus of the Hungarian University of Agriculture and Life Sciences (permit number: MÁB/2-2/2019). The authors declare that all experiments were performed in accordance with the approved guidelines and regulations.

### 2.2. Test Animals

The studies were carried out using the first litters of 40 mature 10–12-month-old European wild rabbits (*Oryctolagus cuniculus*). The rabbits originated from captive wild rabbits. Natural mating was used and the offspring were kept in cages. They were imprinted during the first week of lactation after birth to ensure safe handling by reducing their fear of humans [36,37].

### 2.3. Housing

The lighting period in the building was 16 h (15.4 ± 1.6 h), with artificial lighting provided by lamps on a timer in addition to the light entering through the windows. The animals were housed in individual cages with feeding troughs (40 cm × 25 cm × 31 cm), measuring 60 cm × 60 cm × 45 cm, made of spot-welded wire mesh with hand-operated feeders and hay racks on the front.

### 2.4. Feeding

The rabbits were provided with commercial rabbit feed ad libitum (DE: 10.6 MJ/kg, crude protein: 16.3%, crude fat: 3.8%, crude fiber: 17.7%), hay (100 g/day) and water ad libitum.

### 2.5. Measuring Milk Yield

After birth, we removed the kits from the nest and recorded the number of kits (born alive), individual birth weight and litter weight at 21 days. Measurements were taken on a Sartorius balance to the nearest 0.1 g. Maternal milk production was recorded daily for the first 21 days of lactation [38]. The kits were weighed before and after suckling and the difference between the two weights was used to determine the amount of milk produced. The total amount of milk consumed up to 21 days of age was also calculated.

### 2.6. Sequencing PRLR

DNA extraction from fur samples was performed by excising the fur using 5% Chelex resin [39] according to a standard procedure, resulting in 400 µL of DNA solution of sufficient purity. The DNA solution was adjusted to a concentration of 55 ng/µL. Primers for amplification were designed using the Primer3+ (University of Tartu, Tartu, Estonia) program (primer sequences: 5′ ATAGCTCCCTGAGGCTTGGT 3′ and 5′ TGGGACGTGGAGATCCATTG 3′). The PCR conditions were 95 °C for 10 min, followed by 35 cycles (94 °C for 30 s, 55 °C for 60 s, 72 °C for 90 s), and finally 15 min at 72 °C. The primers were linked with a universal M-13 end, which provided the connection to the sequencing primer. The final volume of the reaction mixture was 20 µL and contained the following components: 2.5 µL genomic DNA solution (55 ng/µL), 10 µL 2× Platinum Superfi MasterMix, 5 µL 5× Enhancer, 1.25–1.25 µL 10µM PRLR-F and PRLR-R primers. The resulting 1210 bp long product was sequenced after silica membrane purification using BigDye Terminator 3.1 sequencing kit (ThermoFisher Scientific, Waltham, MA, USA). The temperature profile of the sequencing reaction was as follows: 96 °C for 3 min, 96 °C for 10 s, 55 °C for 20 s, 60 °C for 1 min for 15 s, then 4 °C. The final volume of the reaction mixture was 10 µL, the composition was 0.8–2 µL sample, 1.4 µL BigDye, M-13 sequencing primer, and distilled water. The products obtained were base sequenced on an ABI 3100 Genetic Analyser (Applied Biosystems, Waltham, MA, USA). The sequence of the 40 maternal PRLR genes was aligned to the corresponding region of the gene bank sequence (ID no. NC_013679.1) using the Clustal Omega program [40] to identify point mutations.

A microsatellite with the sequence ((CTC)6 or (CTC)7 repeat) (from the forward direction) was found in the promoter region (1210 bp), and primers were designed using Primer3 version 4.1.0 software (University of Tartu, Tartu, Estonia). The sequence of the primers was as follows: forward primer 5′TGTTTGGACCACTGACCCTT3′, reverse primer 5′GAGAGCCTCGGTGTCAAATT3′. The final volume of the reaction mixture was 10 µL and contained the following components: 1 µL genomic DNA solution (55 ng/µL), 5 µL 2× Platinum Superfi MasterMix, 2 µL 5× Enhancer, 0.5–0.5 µL 10 µM forward and reverse primers, 1 µL distilled water. The temperature conditions were 95 °C for 15 min, followed by 35 cycles (95 °C for 30 s, 58 °C for 30 s, 72 °C for 45 s), and finally 15 min at 72 °C. A forward primer with NED-fluorescent end-labeling was used for DNA amplification. The fragment length polymorphism analysis, using a LIZ-500 size standard (Life Technologies, Carlsbad, CA, USA) was performed on an ABI 3500 genetic analyzer (Applied Biosystems, Waltham, MA, USA) and results were evaluated by GeneMapper 4.1 software (ThermoFisher Scientific, Waltham, MA, USA).

### 2.7. Statistical Analyses

Related to the genetic diversity, the observed heterozygosity (H_o_), the expected heterozygosity (H_e_), the effective allele number (N_e_) and the Hardy–Weinberg equilibrium were determined using GENALEX version 6.5 [41,42]. Polymorphic Information Content (PIC) was calculated using CERVUS 3.0.7 software [43]. The linkage disequilibrium (LD) values were calculated using DNAsp 5.10 software [44], and the proportion of variance was explained by the number of offspring and by PRLR polymorphisms of the total variance of milk yield was calculated using SPSS 17.0 software (SPSS Inc., Chicago, IL, USA, 2008). This was conducted using a Generalized Linear Model (GLM), where the dependent variable was milk yield, the fixed factors were the four genotypes and microsatellites, and the covariate was litter size. Partial eta squared was calculated to measure the proportion of variance explained by each variable in the model.

## 3. Results

### 3.1. Identification of Point Mutations

**Figure 1:**
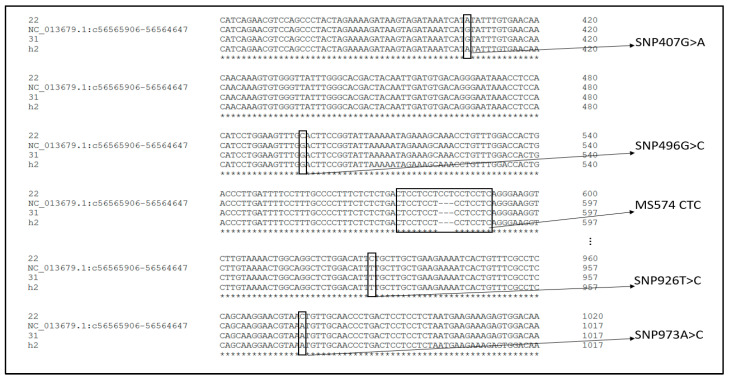
The four point mutations and the microsatellite in the promoter region of PRLR sequence (NC_013679.1 is the reference sequence from the GenBank, the * indicates identical nucleotides in the sequences.)

Sequencing of the promoter region of the PRLR gene identified four point mutations located at SNP1-407G > A, SNP2-496G > C, SNP3-926T > C and SNP4-973A > C. In addition to the point mutations, a microsatellite was detected at position 574 (Figure 1 and Figure S1).

The Table S1 contains the row data of the experiment. Table 1 shows the distribution of observed genotypes, observed heterozygosity (H_o_), expected heterozygosity (H_e_), effective allele size (N_e_) and PIC value. Examination of the distribution of genotypes indicates that they are in Hardy–Weinberg equilibrium for the 926T > C and 973A > C SNPs (*p* > 0.05), while they are not in equilibrium for the other two SNPs. PIC values show that the rabbit population presents moderate polymorphisms for each of the point mutations.

### 3.2. The linkage between Point Mutations

Table 2 shows the relationships between SNPs. Based on our results, all four SNP pairs showed significant linkage disequilibrium (LD) (linked inheritance). The four SNPs in the population were segregated into the following four genotypes: AACCCCCC, GGGGTTAA, AAGGTTAC, and GGGGTCAC.

Several factors significantly affected milk production, such as the number of kits and microsatellites found in PRLR, as well as the genotypes constituted by SNPs (Table 3).

Regarding genotypes, the GGGGTTAA genotype in homozygous form showed higher milk production (1564.7 ± 444.7 g) compared to the other three genotypes (AACCCCCC 1399.1 ± 326.8 g; GTGACCTT 1403.8 ± 517.1 g; GGGGTCAC 1220.0 ± 666.2 g). The interaction between microsatellite and SNP genotypes was non-significant.

**Figure 2:**
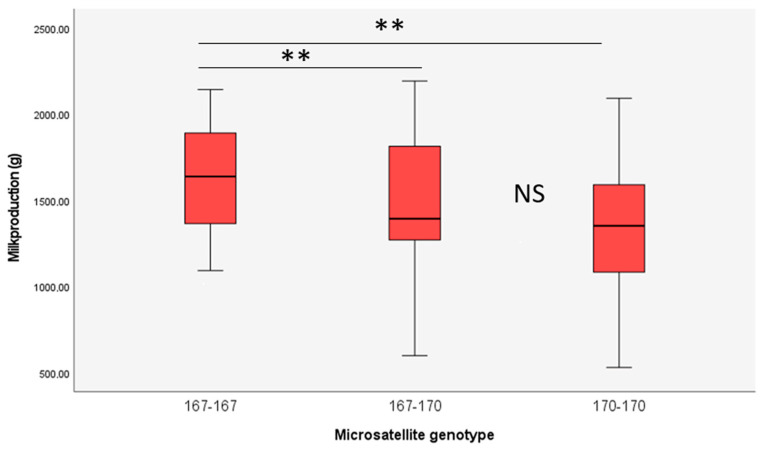
Distribution of milk production in groups of mothers with different microsatellite genotypes. 167/167 and 170/170 indicate the fragment lengths of the two homozygous genotypes, 167/170 indicates the fragment lengths of the heterozygous genotype (** indicates significant difference at *p* < 0.01 level).

The distribution of milk production according to microsatellite genotypes are shown in Figure 2. The short repeat, a 167 base fragment, resulted in significantly (*p* = 0.003) higher milk production (1623.8 ± 525.1 g) compared to the long repeat (170 bases, 1300.4 ± 458.6 g) and the heterozygous form (167/170) (1460.4 ± 411.5 g) (GLM model). The difference between the heterozygous form and the long repeat was not significant.

## 4. Discussion

We identified four point mutations (SNP1-407G > A, SNP2-496G > C, SNP3-926T > C and SNP4-973A > C) and one microsatellite at position 574 by sequencing the promoter region of the PRLR gene of our European wild rabbit population. Our population is a cross of Hungarian wild rabbits (from the Bugac area) and Slovakian wild-caught wild rabbits. Therefore, we expected high diversity and heterozygosity values. However, the analysis of genotype distributions resulted in a Hardy–Weinberg equilibrium (*p* > 0.05) only for SNPs 973A > C and 339G > A, in the other two SNPs a complete absence of heterozygotes was detected. H_o_ values were lower than H_e_ values, suggesting inbreeding. This was probably due to the limited number of samples, despite the fact that the individuals in the study were from populations of two different areas. This is confirmed by the presence of significant pairwise disequilibrium (LD) of SNPs and by their segregation into four genotypes in our population (AACCCCCC, GGGGTTAA, AAGGTTAC, GGGGTCAC). Based on PIC values, all point mutations can be classified as moderately informative markers [45]. Polymorphisms in the PRLR gene have not yet been studied in rabbits; therefore, our genetic diversity data, when compared to genetic diversity data for polymorphisms in the progesterone receptor gene in domesticated rabbit lines, show similar values [46].

Our results show that genetic background has a strong influence on milk production by rabbits. The homozygous genotype (GGGGTTAA 1564.7 ± 444.7 g) and the short repeat of the microsatellite (167 bp 1623.8 ± 525.1 g) from the genotypes in the regulatory region of the PRLR gene resulted in significantly higher milk production (GLM model, (*p* = 0.003)). The role of the PRL hormone is extremely diverse, its best-known impact is made on the mammary glands [18]. The hormonal requirements for initiating and maintaining milk production vary between species. However, they have one factor in common; PRL is the hormone primarily responsible for milk production, milk protein [47], lactose [48] and lipid synthesis [49]. PRL, in cooperation with its receptors (PRLR), has a number of effects and a very complex regulation [18]. Therefore, mutations in the PRLR gene may affect the function of PRL. This may explain the observed association with milk yield. In rabbits, the relationship between the PRLR gene and milk production has not yet been investigated, although previous studies have suggested that the genetic background has an important role [34,35]. The milk yield of Holstein dairy cattle (*Bos taurus*) has been associated with polymorphisms in exons 3 and 7 of the PRLR gene, the results of these studies suggest that the PRLRE3 and PRLRE7 loci of the PRLR gene are useful genetic markers in milk selection programs [21,50]. In Finnish Ayrshire dairy cattle, polymorphisms in two other regions of the PRLR gene (exons 9 and 10) were found by QTL analysis. These polymorphisms were significantly associated with both milk yield and protein and fat content of milk [20]. In addition (*Capra aegagrus hircus*), four SNPs in the PRLR gene (g.40452T > C, g.40471G > A, g.61677G > A, g.61865G > A) were described in goats. Similar to the results of our study, the group of individuals having the homozygous (TTAAGGGG) combination of haplotypes had significantly higher milk yields [28].

Although microsatellite markers are generally considered to be neutral markers, they may affect gene activity when located in promoter regions [51]. Our results suggest that CTC repeats in the promoter region affect PRLR gene function. The repeats were present five or six times in the promoter sequence of the gene, and the length of polymorphisms showed a significant difference in milk production during the first 21 days of lactation of wild rabbits. In the 5′ flanking region, as in the promoter region, DNA polymorphisms can affect the pace of transcription and thus the formation of protein products. In several cases, polymorphisms in or near the 5′ flanking region of genes in farm animals have been found to modify production traits [52]. Depending on the number of repeats, microsatellites located in the 5′ flanking region of the genes modified the expression of the mouse GH receptor gene [53], the goat growth hormone gene [54] and the tilapia PRL gene [55].

Our study investigated the association between the regulatory region of the PRLR gene and milk production. Further studies would be needed with differentially selected domestic breeds (and lines) to elucidate the regulatory effect of microsatellites on milk production. This microsatellite could be a potential marker to develop marker-based selection (MAS). An important step would be to investigate the coding regions of the PRLR gene, as this would provide a more complex picture of the genetic regions influencing rabbit milk production.

## 5. Conclusions

Our results show that the wild rabbit is a suitable model for studying the relationship between genetic variation and production parameters. The association between polymorphisms in the PRLR gene and milk production in our small model population allows the development of marker-based selection to improve milk production in rabbit species. This would require further studies with differently selected domestic breeds to investigate the effect of genotypes and microsatellites on milk production.

## Figures and Tables

**Table 1:** Genotypic distribution and genetic diversity in four SNPs located on the PRLR.

SNP	Observed Genotype						H_o_	H_e_	HWE		N_e_	PIC
									χ^2^	Prob.		
407G > A	GG	28	GA	0	AA	12	0.000	0.425	40.000	<0.001	1.724	0.332
496G > C	GG	19	GC	0	CC	21	0.000	0.505	40.000	<0.001	1.995	0.374
926T > C	TT	21	TC	15	CC	4	0.375	0.415	0.287	0.592	1.694	0.326
973A > C	AA	21	AC	15	CC	4	0.375	0.415	0.287	0.592	1.694	0.326

H_o_: observed heterozygosity, H_e_: expected heterozygosity, N_e_: effective allele size and PIC: Polymorphism information content, HWE: the Hardy-Weinberg equilibrium, Prob.: probability.

**Table 2:** Allele and haplotype frequency distribution and linkage disequilibrium in the case of tested SNPs.

	Allele Frequency		Haplotype Frequency		D’	r	χ^2^	*p*
SNP1-2	G	0.300	GG	0.3	0.323	0.688	18.947	<0.001
	A	0.700	GA	0.175				
	G	0.475	CG	0				
	C	0.525	CA	0.525				
SNP1-3	G	0.300	TG	0.225	0.233	0.745	22.185	<0.001
	A	0.700	TA	0.0875				
	T	0.288	CG	0.075				
	C	0.713	CA	0.6125				
SNP1-4	G	0.300	AG	0.225	0.233	0.745	22.185	<0.001
	A	0.700	AA	0.0875				
	A	0.288	CG	0.075				
	C	0.713	CA	0.6125				
SNP2-3	G	0.475	TG	0.3125	0.310	0.907	32.894	<0.001
	C	0.525	TC	0				
	T	0.288	CG	0.1625				
	C	0.713	CC	0.525				
SNP2-4	G	0.475	AG	0.3125	0.310	0.907	32.894	<0.001
	C	0.525	AC	0				
	A	0.288	CG	0.1625				
	C	0.713	CC	0.525				
SNP3-4	T	0.288	AT	0.23125	0.228	1.000	40.000	<0.001
	C	0.713	AC	0.08125				
	A	0.288	CT	0.08125				
	C	0.713	CC	0.60625				

D’: distance from equilibrium, r: correlation coefficient, χ^2^: Chi-square value, *p*: significance level.

**Table 3:** Association of milk production (21-day total milk volume) with polymorphisms of PRLR (Generalized Linear Model (GLM), the number of kits were covariant.

	df	Milk Production			
		Mean Square	F	*p*	Partial Eta Square
Corrected model	7	603,986.881	5.419	0.000	0.542
Intercept	1	1,659,510.225	14.888	0.001	0.318
Number of kits	1	1,239,088.433	11.116	0.002	0.258
Genotype	3	487,348.278	4.372	0.011	0.291
MS574	2	758,532.337	6.805	0.003	0.298
MS574 × genotype	1	2304.989	0.021	0.887	0.001
Error	32	111,466.214			

Generalized linear model: covariates: cortisol and the number of kits, fix factors: the length of the microsatellite and the genotypes.

## Data Availability

The raw data of the study is available in the Supplementary material (Supplementary Material Figure S1 and Table S1).

## Supplementary Materials

The following supporting information can be downloaded at: https://www.mdpi.com/article/10.3390/ani13040671/s1, Figure S1: Sequence of the investigated promoter section of PRLR gene in wild rabbit; Table S1: The raw data of the investigation.
