# Supplementary material for: The Role of PRLR Gene Polymorphisms in Milk Production in European Wild Rabbit (Oryctolagus cuniculus)
Source: Animals (Basel). 2023 Feb 15;13(4):671. doi: 10.3390/ani13040671 (PMC9951758; doi:10.3390/ani13040671)
Supplement: Supplementary file 1 [file animals-13-00671-s001.zip › Figure S1.pdf]

>wild rabbit PRLR gene promoter sequence

ATAGCTCCCTGAGGCTTGGTAAGCAGCAACTACCTGCTCAACGCCAGGTGCCCCA
GAGCAGCAGTCAAATCAAAGGGGAAGCCCAGGGTTCCTGCAGCTGTTTTTTGAAA
CTCAATAGCCTTTATTCAAAGTATCTCCCTAGCCACCCTCCAGGTTACATAACAAT
CAGGTGCAATATTAATTAGTGACATCAAACAGACACTAGCTACCTACTCATTCAA
GGATAACTCTTTGATTCAGGCAACTGCTACTACTATTTGTTTCAGTACTTTTGCAA
GAGAAAAAGTGAGTGGTGGTTGTGTGTGGTTTTGTTAATACTAAAACCAAACCAA
AAAAAAAAAAAAAAGGAAAAAGGAAAAAGCATCAGAACGTCCAGCCCTACTAGA
AAAGATAAGTAGATAAATCATgTATTTGTGAACAACAACAAAGTGTGGGTTATTT
GGGCACGACTACAATTGATGTGACAGGGAATAAACCTCCACATCCTGGAAGTTTG
gACTTCCGGTATTAAAAATAGAAAGCAAACCTGTTTGGACCACTGACCCTTGATTT
TCCTTTGCCCCTTTCTCTCTGActcctcctcctcctcAGGGAAGGTCAGACTGAAATCCCAG
AGCCCCCAACCCCCCATCACAAATATTGTCAAGCTGCCATTCAGTGTTGCTGGGA
AATAAGAAATTTGACACCGAGGCTCTCTCCATGGCTTGGCAGCAAAGAGAAATAT
CATCTTTGAGGTCATTCGACCGATTCAGACATTATTATTTCTGTTTTGTCTGTGTTT
TCTGCTACAGTGTCAACGCAAGATGCGTGGCCTTGATCGTGTAGATCTGATCTGG
GGAGAATCCGTGAATTCTTCACATCTTCGCTGCATTCCGGCTCCTCCGACCGCGTC
CCTCACCTTGTAAAACTGGCAGGCTCTGGACATTtTGCTTGCTGAAGAAAATCACT
GTTTCGCCTCCAGCAAGGAACGTAAaTGTTGCAACCCTGACTCCTCCTCTAATGAA
GAAAGAGTGGACAAGTGCACCGAGTTGAGCTGCTGCTCGCAGAAGCCACCGGGC
TGCCCACGGAAGCTGAAAGCCCCAGACAGCACTGCTCCTGGGCTGGGCTTCCCGC
CCTGGCCTTTCTGTCGTACTAGCTCTTCTCCCCCTCCTTTCTGGATTTTACCGGCTG
TTCGCGAAACAGCTTTCCACACAATGGATCTCCACGTCCCA
