# Supplementary material for: The Role of PRLR Gene Polymorphisms in Milk Production in European Wild Rabbit (Oryctolagus cuniculus)
Source: Animals (Basel). 2023 Feb 15;13(4):671. doi: 10.3390/ani13040671 (PMC9951758; doi:10.3390/ani13040671)
Supplement: Supplementary file 1 [file animals-13-00671-s001.zip › Table S1.pdf]

Doe ID	973AC	926TC	496GC	407GA	Genotype	Msat	Litter size	Milkproduction
1	1	1	1	1	1111	3	7	1686,60
2	2	2	3	3	2233	2	4	1093,00
3	1	1	1	1	1111	3	3	741,02
4	2	2	3	3	2233	2	6	1812,54
5	1	1	1	1	1111	3	4	914,00
6	1	1	1	1	1111	3	5	1571,88
7	1	1	1	1	1111	3	2	1034,00
8	1	1	1	1	1111	3	5	1352,09
9	2	3	3	1	2331	2	6	2098,97
10	2	2	3	3	2233	2	5	1530,00
11	2	2	3	3	2233	2	8	1392,76
12	3	3	3	3	3333	1	6	1637,60
13	3	3	3	3	3333	2	6	1387,50
14	1	1	1	1	1111	3	4	1254,00
15	2	2	3	1	2231	2	3	1237,00
16	1	1	1	1	1111	3	6	1828,00
17	2	2	3	3	2233	2	3	598,00
18	1	1	1	1	1111	3	5	1366,00
19	1	1	1	1	1111	3	8	1608,00
20	1	1	1	1	1111	3	4	1291,00
21	1	1	1	1	1111	3	5	1333,97
22	1	1	1	1	1111	3	6	1556,00
23	2	2	3	1	2231	2	6	1285,00
24	2	2	3	1	2231	2	5	1325,91
25	2	2	3	1	2231	3	4	529,00
26	2	2	3	1	2231	2	5	2192,00
27	3	3	3	3	3333	1	6	2142,00
28	2	2	3	1	2231	2	5	1814,00
29	1	1	1	1	1111	3	3	1486,00
30	1	1	1	1	1111	3	3	1132,00
31	1	1	1	1	1111	3	2	1221,00
32	1	1	1	1	1111	3	4	1716,00
33	2	2	3	1	2231	2	2	1444,00
34	1	1	1	1	1111	3	4	1650,00
35	3	3	3	3	3333	1	3	1092,00
36	2	2	3	3	2233	2	6	2079,00
37	2	2	3	3	2233	2	3	1255,00
38	1	1	1	1	1111	3	5	1542,00
39	1	1	1	1	1111	3	5	1005,00
40	1	1	1	1	1111	3	4	2092,00
